# Surgical skill level classification model development using EEG and eye-gaze data and machine learning algorithms

**DOI:** 10.1007/s11701-023-01722-8

**Published:** 2023-10-21

**Authors:** Somayeh B. Shafiei, Saeed Shadpour, James L. Mohler, Farzan Sasangohar, Camille Gutierrez, Mehdi Seilanian Toussi, Ambreen Shafqat

**Affiliations:** 1 Intelligent Cancer Care Laboratory, Department of Urology, Roswell Park Comprehensive Cancer Center, Buffalo, NY 14263 USA; 2 Department of Animal Biosciences, University of Guelph, Guelph, ON N1G 2W1 Canada; 3 Department of Urology, Roswell Park Comprehensive Cancer Center, Buffalo, NY 14263 USA; 4 Mike and Sugar Barnes Faculty Fellow II, Wm Michael Barnes and Department of Industrial and Systems Engineering at Texas A&M University, College Station, TX 77843 USA; 5 Obstetrics and Gynecology Residency Program, Sisters of Charity Health System, Buffalo, NY 14214 USA

**Keywords:** Blunt dissection, Retraction, Burn dissection, Hysterectomy, Cystectomy, Nephrectomy, Robot-assisted surgery, Expertise level

## Abstract

The aim of this study was to develop machine learning classification models using electroencephalogram (EEG) and eye-gaze features to predict the level of surgical expertise in robot-assisted surgery (RAS). EEG and eye-gaze data were recorded from 11 participants who performed cystectomy, hysterectomy, and nephrectomy using the da Vinci robot. Skill level was evaluated by an expert RAS surgeon using the modified Global Evaluative Assessment of Robotic Skills (GEARS) tool, and data from three subtasks were extracted to classify skill levels using three classification models—multinomial logistic regression (MLR), random forest (RF), and gradient boosting (GB). The GB algorithm was used with a combination of EEG and eye-gaze data to classify skill levels, and differences between the models were tested using two-sample *t* tests. The GB model using EEG features showed the best performance for blunt dissection (83% accuracy), retraction (85% accuracy), and burn dissection (81% accuracy). The combination of EEG and eye-gaze features using the GB algorithm improved the accuracy of skill level classification to 88% for blunt dissection, 93% for retraction, and 86% for burn dissection. The implementation of objective skill classification models in clinical settings may enhance the RAS surgical training process by providing objective feedback about performance to surgeons and their teachers.

**Supplementary Information:**

The online version contains supplementary material available at 10.1007/s11701-023-01722-8.

## Introduction

Robot-assisted surgery (RAS) has revolutionized surgical procedures by providing benefits, such as increased precision, reduced surgical trauma, and improved patient outcomes [1]. As surgeons increasingly turn to RAS for procedures, such as cystectomy, hysterectomy, and nephrectomy, new skills must be acquired to operate the robot and perform surgical procedures. Safe and effective performance of surgical subtasks, such as dissection and retraction, requires a high level of skill and expertise in RAS. Objective measurement and evaluation of these skills are necessary to train and evaluate RAS surgeons, ensuring safety and effectiveness.

Objective and consistent assessment of surgical skills is important, but current surgical practice protocols lack such methods. Prior research has proposed objective techniques to evaluate skills using physiological data, including brain activity, eye movement, kinematics, and surgical videos [2–4]. These methods have shown promising results for objectively assessing RAS skills; however, they also have limitations, such as only testing basic tasks in a dry lab with a small number of participants, introducing biases, or creating models that are computationally expensive and cannot be integrated into surgical robot systems.

One potential approach for objectively evaluating surgical skills in RAS is the use of electroencephalogram (EEG), eye-gaze features, and machine learning algorithms. EEG is a noninvasive technique that measures the electrical activity of the brain and has been used in various studies to investigate the cognitive processes involved in performing surgical tasks [5]. Machine learning algorithms have been proposed as useful tools for classifying various levels of surgical skills based on features extracted from physiological data during RAS tasks [6, 7] (Table 1). It has been shown that eye-gaze features are significantly different for inexperienced, competent, and experienced participants performing RAS subtasks in the operating room [8].

**Table 1:** State-of-the-art studies proposing surgical skill classification models for RAS in the operating room

Study	Data	Trials	Subjects	Task	Number of skill levels	Algorithm	Accuracy
Chen et al., 2020 [6]	Kinematics	68	17	Needle handling/targeting, needle driving, suture cinching	2	AdaBoost, gradient boosting, and random forest	77.40%
Lee et al., 2020 [7]	Endoscopic videos	4	12	Bilateral axillo-breast approach robotic thyroidectomy	3	support vector machine (SVM), and random forest	83%
Current study	EEG and eye gaze data	212 blunt dissection, 1017 retraction, and 324 burn dissection	11	Hysterectomy, cystectomy, and nephrectomy	3	Multinomial logistic regression, random forest, and gradient boosting,	88%, 93%, and 86% for blunt dissection, retraction, and burn dissection, respectively

Several EEG features have been proposed and compared across surgical skill levels that have demonstrated significant differences between experts and novices, or among novice, intermediate, and expert categories [2, 5]. However, in most of these studies, the ability of neuromonitoring findings to classify subjects accurately by skill level was not analyzed. Therefore, further investigation is warranted in this area.

This study explored the classification of surgical skill levels in RAS performing subtasks using EEG, eye-gaze features, and three machine learning algorithms.

## Methods

This study was approved by the Institutional Review Board (IRB: I-241913) and Institutional animal care and use committee approval (IACUC 1179S) of Roswell Park Comprehensive Cancer Center. The IRB granted permission to waive the need for written consent. Participants were given written information about the study and provided verbal consent.

### Participants

**Fig. 1:**
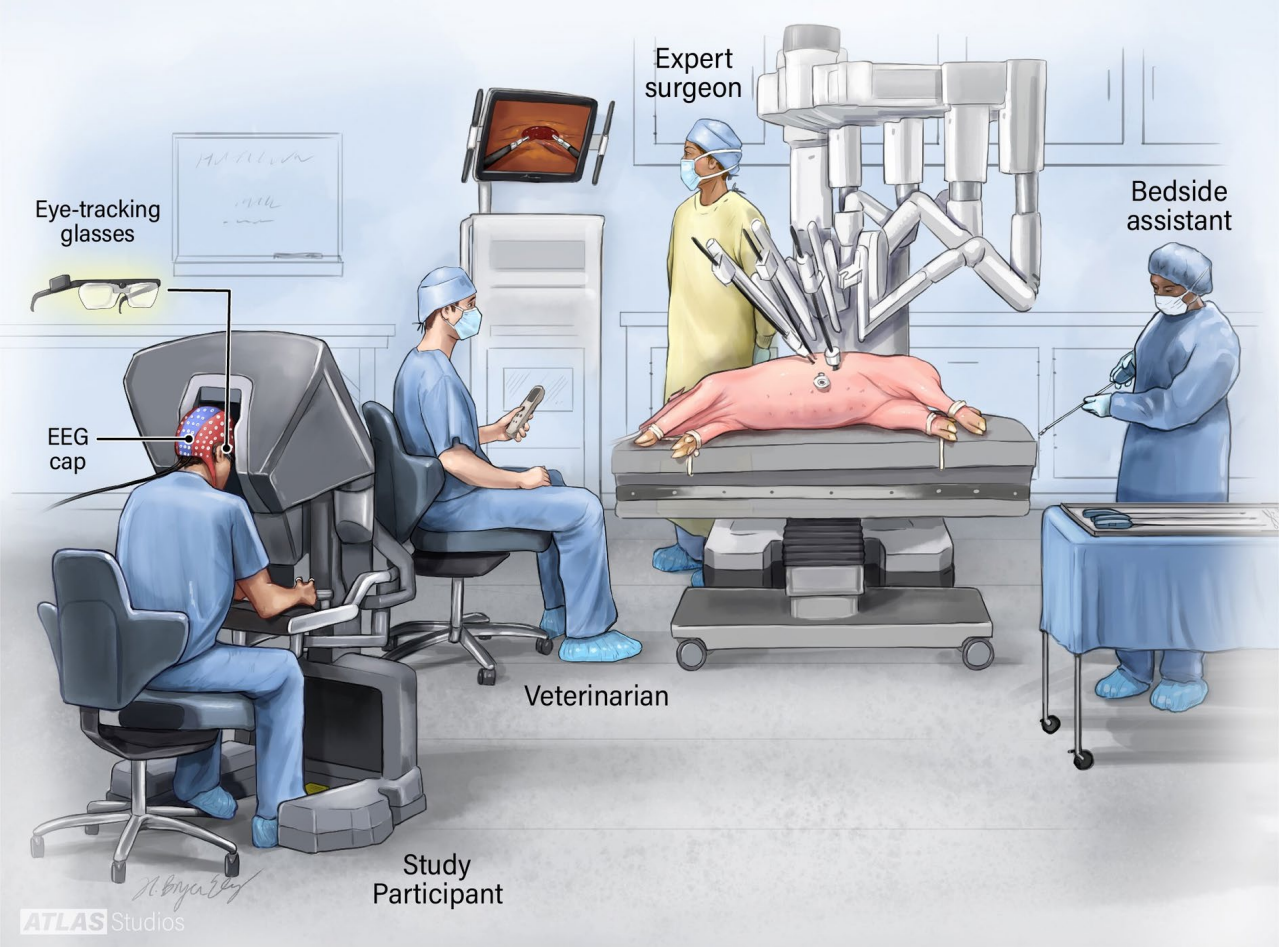
Experimental setup. Representation of participant wearing EEG headset and eye tracking glasses performing surgical tasks using the da Vinci robot on pig in the operating room

Eleven participants (10 males, 1 female), aged 42 ± 12 years, including two residents, four fellows, and five surgeons, performed 11 hysterectomies, 11 cystectomies, and 21 nephrectomies using the da Vinci surgical robot on live pigs (Fig. 1).

### Data recording

EEG data recorded via the 124-channel AntNeuro^®^ EEG system (500 Hz) and eye-gaze data via Tobii^®^ eyeglasses (50 Hz).

### Subtask extraction

EEG data were extracted for 324 blunt dissection subtasks, 1508 retraction subtasks, and 542 burn dissection subtasks, as well as eye gaze data for 212, 1017, and 324 subtasks, respectively [9].

### Actual skill levels

An expert RAS surgeon with more than two decades of experience watched operation videos and assessed the surgical expertise level of each participant in performing each subtask using the modified Global Evaluative Assessment of Robotic Skills (GEARS) assessment tool [10] at three levels: inexperienced, competent, and experienced.

### Definition of EEG features

EEG signals were processed to remove artifacts using the signal processing steps detailed in our previous publication [11]. Each EEG channel was assigned to a specific area of the brain called Brodmann’s area (BA). EEG features were extracted from different brain areas [11–23]. The brain stores information in specific areas when new skills are acquired [24]. Practice and training results in changes in the functional brain network [24]. These changes were quantified by extracting features, such as strength, search information, temporal network flexibility, integration, and recruitment [11].

These features provide an understanding of how the brain processes information during surgery. For example, search information indicates how efficiently information is passed between different parts of the brain [18, 25], whereas strength indicate how well different brain areas communicate with one another. Flexibility provides an understanding of how the brain changes over time in response to different demands [21], whereas integration describes how different parts of the brain work together over time [23]. Recruitment refers to the activation of a specific brain area that forms interconnected networks when performing cognitive or behavioral tasks. This recruitment pattern can provide important insights into the underlying neural mechanisms of different cognitive functions and can help understand how the brain processes information and generates behavior [26]. These features were calculated for 21 different areas of the brain [27], and 105 features were extracted.

### Definition of eye-gaze features

Eye-gaze features, which include average pupil diameter, entropy of pupil diameters, total length of pupil trajectory, fixation rate, and saccade rate were extracted [28]. These features are often used in eye-tracking studies to gain insight into cognitive processes, such as attention, perception, and decision-making [29]. The average pupil diameter indicates arousal or interest, entropy measures variation in pupil size, length of pupil trajectory measures the distance covered, fixation rate measures fixation time, and saccade rate measures the frequency of rapid eye movements between fixations.

### Machine learning models for skill level classification using EEG features

The extracted EEG features for each subtask and the actual surgical skill levels were used as inputs for the gradient boosting classification (GB), Random Forest (RF), and Multinomial Logistic Regression (MLR) classification algorithms to develop models for classifying the three skill level classes.

Twenty percent of the samples were randomly chosen and used as the test set. The remaining 80% were used to train and validate the model. The hyperparameters of each model (Supplement 1) were optimized using grid search technique and stratified five fold cross-validation that was repeated five times. The synthetic minority over-sampling technique was applied to the training sets to address the issue of imbalanced data across different classes [30]. The model training and testing was repeated 30 times and average performance metrics were reported. Details on training these models and improving their performance are provided in Supplement 1.

### Machine learning models for skill level classification using EEG and eye-gaze features

EEG and eye-gaze features and actual surgical skill levels were inputted into the GB classification to classify skill levels for each subtask. The same process was used to develop GB models with a combination of EEG and eye-gaze features. Feature importance was determined using permutation-based methods.

### Evaluation of machine learning models

True positives (TP) were the samples, where the model correctly predicted the positive class, while false positives (FP) were the samples, where the model predicted the positive class, but the actual class was negative. The performance of the developed models in classifying the surgical skill levels of participants was evaluated using various statistical measurements. These included:

- Precision: The ratio of TP and (TP + FP).
- Recall: The ratio of TP and (TP + FN).
- Average accuracy: Ratio of the sum of correct predictions to the total number of predictions.
- F-score: A measure of a model's accuracy that combines precision and recall into a single metric, ranging from 0 to 1, where a higher value indicates better performance.
- Receiver Operating Characteristic (ROC) curves and area under the curve (AUC) are used to evaluate classifier performance. The ROC is a graph of the true positive rate against the false positive rate at different threshold values. The AUC is a numerical value ranging from 0 to 1 that represents the probability of the classifier correctly identifying a randomly chosen positive or negative example. An AUC of 0.5 represents a random classifier, while an AUC of 1 represents a perfect classifier.
- Confusion matrix: This matrix was used to evaluate the performance of the machine learning model by comparing the actual and predicted values.

Two-sample *t* tests were applied to pairs of accuracy results for 30 runs of each model to assess the statistical significance of any observed differences between the models. The Bonferroni *p* value correction was applied to adjust the *p* values resulting from conducting pairwise comparisons among the three models.

## Results

### Skill levels classification models in conducting blunt dissection using EEG features

Table 2 presents the results of the skill level classification in conducting blunt dissection subtasks. The accuracies of the RF and MLR models were similar (*p* value = 0.34). However, the accuracy of the GB model was significantly better than that of the MLR model (*p* value = 1 × 10^–3^). The accuracy of the GB model was significantly better than that of the RF model (*p* value = 2 × 10^–4^).

**Table 2:**
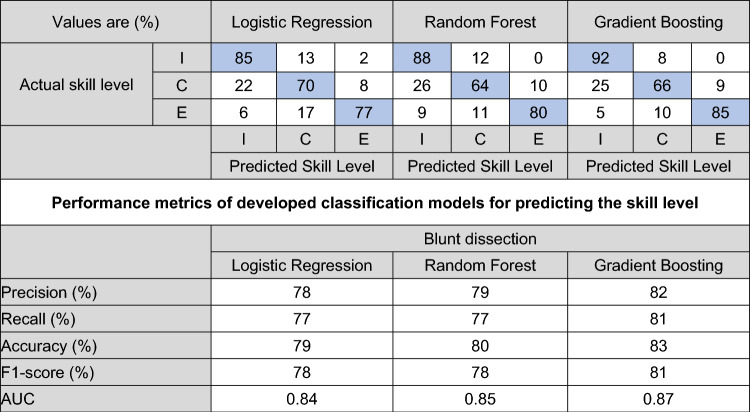
Confusion matrix for the classification of skill levels—inexperienced (I); competent (C); experienced (E)—of participants performing blunt dissection using three machine learning models: multinomial logistic regression, random forest, and gradient boosting

### Skill levels classification models in conducting retraction using EEG features

Table 3 presents the results of the skill level classification. The accuracy of the RF model was significantly better than that of the MLR model (*p* value = 4.8 × 10^–15^), and the accuracy of the GB model was significantly better than that of the MLR model (*p* value = 2.8 × 10^–16^). The accuracy of the GB model was significantly better than that of the RF model (*p* value = 3 × 10^–3^).

**Table 3:**
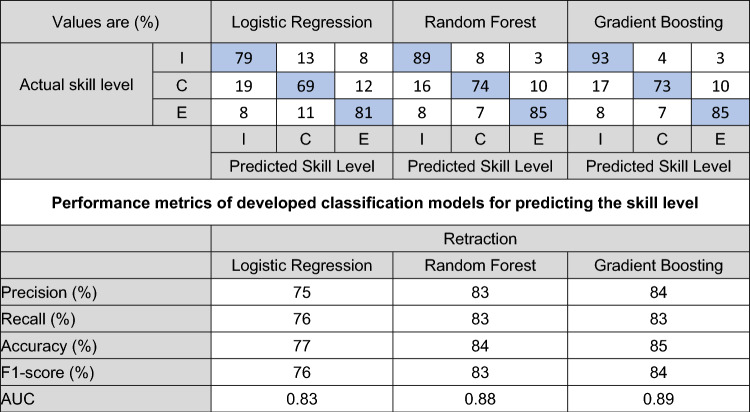
Confusion matrix for the classification of skill levels—inexperienced (I); competent (C); experienced (E)—of participants performing retraction using three machine learning models: multinomial logistic regression, random forest, and gradient boosting

### Skill levels classification models in conducting burn dissection using EEG features

Table 4 displays the confusion matrix for the classification of skill levels. The accuracy of the RF model was significantly better than that of the MLR model (*p* value = 5 × 10^–3^), and the accuracy of the GB model was significantly better than that of the MLR model (*p* value = 1.4 × 10^–6^). The accuracy of the GB model was significantly better than that of the RF model (*p* value = 1.4 × 10^–5^).

**Table 4:**
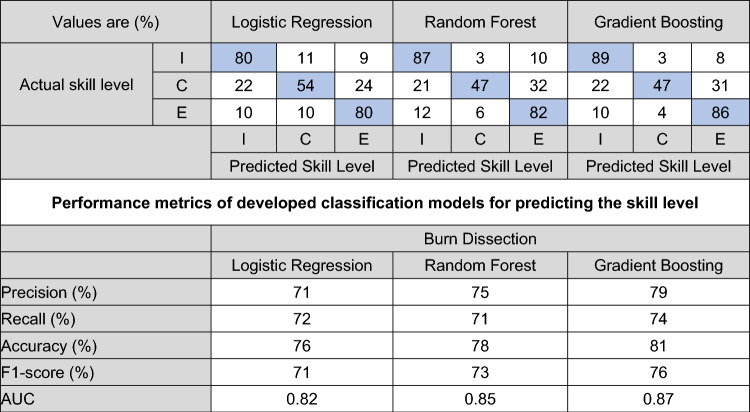
Confusion matrix for the classification of skill levels—inexperienced (I); competent (C); experienced (E)—of participants performing burn dissection using three machine learning models: multinomial logistic regression, random forest, and gradient boosting

### Classification models using EEG and eye-gaze features

Table 5 shows the confusion matrix for classifying skill levels in blunt dissection, retraction, and burn dissection using the GB model.

**Table 5:**
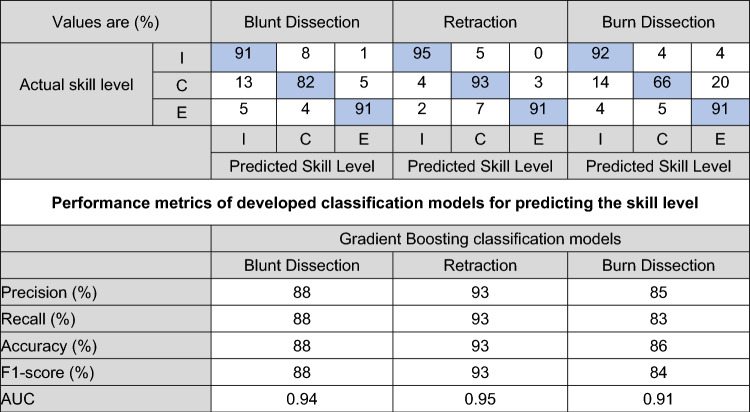
Confusion matrix for the classification of skill levels—inexperienced (I); competent (C); experienced (E)—of participants performing blunt dissection (number of samples: 212), retraction (number of samples: 1017), and burn dissection (number of samples: 324) using EEG and eye-gaze features and gradient boosting

The most significant features in the classification model for blunt dissection subtask were the length of the dominant eye’s pupil trajectory, average recruitment, and integration of channels in several brain areas. For retraction, the top features were the length of both the dominant and nondominant eyes’ pupil trajectories and the average recruitment and integration of channels in several brain areas, while for burn dissection, the top features were the length of both the dominant and nondominant eyes’ pupil trajectories, entropy of the nondominant eye’s pupil diameter, and average integration of channels in different brain areas.

## Discussion

Improved approaches are needed to assess surgical skills, enhance training, and ensure patient safety. Manual methods for skill assessment have proven to be simple to use, but they require a panel of experts who may be biased. Objective methods for skill assessment allow for individualized skill development, ultimately leading to improved surgical outcomes. Developing an effective method for evaluating surgical skills is essential to reduce medical errors. While some studies suggest that experience alone can be used to determine skill level, as demonstrated by surgeon operating volume, this approach has limitations. Surgeons may excel in some tasks but not in others, or they may perform certain activities poorly despite performing many operations. However, objective skill evaluation of RAS in clinical settings remains challenging, despite recent advances in RAS.

EEG features were extracted from different areas of the brain to understand information processing across the brain, how efficiently different parts of the brain communicate with each other, how the brain changes over time in response to different demands, how different parts of the brain work together over time, and the activation of specific brain areas that form interconnected networks when performing cognitive or behavioral tasks. Eye gaze features were extracted, because eye gaze patterns infer participants’ focus of attention and level of engagement with the task. Machine learning models were developed using the extracted features and actual skill levels.

The results suggest that the GB model shows promise in accurately predicting surgical skill levels, particularly when combined with EEG and eye-gaze features (Table 5). The results indicated that the models could predict the skill levels of the participants with high precision, recall, accuracy, and F1-score rates. The AUC values ranged from 0.91 to 0.95, which suggests that the models performed well in discriminating between different skill levels. These findings suggest that a multimodal system that incorporates both EEG and eye-tracking data is necessary to achieve more accurate skill level prediction. Various studies have been conducted to propose RAS skill classification models in the OR using kinematic and video data (Table 1). The current study used EEG and eye gaze data and developed three machine learning models to classify the three surgical subtasks into three skill levels. The results of the current study showed high skill prediction accuracies (88%, 93%, and 86% for blunt dissection, retraction, and burn dissection, respectively) compared to other studies, which demonstrated accuracy rates ranging from 77.4 to 83%. The accuracy of the developed classification models (Table 5) outperformed the state-of-the-art models for RAS skill classification in clinical settings (Table 1) [31].

These results suggest that eye movements and brain activity in specific areas play important roles in the surgical performance of all three subtasks. Specifically, the length of eye’s pupil trajectories is an important factor for all subtasks, and the entropy of the nondominant eye’s pupil diameter is a significant factor in burn dissection. In addition, the recruitment and integration of channels in several brain areas are important for all three subtasks, indicating that cognitive factors, such as attention and decision-making, are crucial in surgical performance.

The present findings may contribute to the development of more accurate and efficient models for surgical skill assessment, which can ultimately improve patient outcomes and enhance surgical training. These results are important, because accurately predicting the skill level of medical professionals in performing surgical procedures can improve patient outcomes and safety. By identifying individuals who may need additional training or support, hospitals and medical institutions can ensure that their staff is adequately prepared and skilled in performing surgical procedures. The use of machine learning models can facilitate this process by providing a fast, accurate, and objective assessment of skill level.

### Strengths of this study

Compared to previous studies, this research has several strengths. First, it focuses on predicting skill levels in individual subtasks instead of the entire surgical procedure, offering more comprehensive insights. Second, actual skill levels assessed by an expert RAS surgeon were used instead of unreliable measures, such as years of experience. Third, the study employed various machine learning models to ensure robustness. Fourth, multimodal system data were incorporated for a comprehensive view of skill assessment. Fifth, three skill levels were considered for detailed analysis, and finally, real operations in the operating room were evaluated, making the results more applicable to real-world scenarios.

### Practical implications of results in RAS training

The machine learning models that were developed may determine whether a RAS trainee needs to practice a specific subtask, which makes the learning process faster and less expensive, because the trainee can focus on specific areas that need improvement instead of repeating the entire operation. This approach can result in more RAS trainees being accepted into training programs and completing them faster. Hospitals will also benefit from this approach, because RAS has shorter hospital stays and fewer complications than conventional surgical methods. The skill classification models that were developed provide a basis for the objective evaluation of RAS skills and performance, which can provide trainees with more useful, immediate, and perhaps more accurate feedback. This could lead to the standardization of RAS training programs for trainees rather than relying on the opinions of an expert panel. Moreover, experienced open surgeons wanting to develop RAS skills will benefit from the proposed approach. This group has already developed significant surgical skills but may lack experience in using RAS.

### Limitations of this study and future research

Despite the promising results of this study, some limitations should be addressed in future research. The study involved only 11 participants and the assessment of GEARS metrics was conducted by only one expert RAS surgeon (J.L.M.). To validate the developed models, it is necessary to include more participants with various specialties from different training programs as well as assessments from more expert RAS surgeons.

Based on the findings of this study, the following research steps could be involved: 1) including assessments from more expert RAS surgeons from different institutes, 2) developing automatic subtask extraction models, and 3) expanding the models developed in this study by incorporating data from more participants with diverse specialties and RAS experience from different institutes. Developing a fully automatic model that uses EEG and eye-gaze data, extracts subtasks, and detects the skill level and score of GEARS metrics for each subtask could enhance the RAS training. Such a model can provide trainees with feedback on their skills and performance and surpass what one-on-one teaching by an expert and proficiency evaluations by a panel of experts offers.

## Conclusions

The results demonstrated the potential of using EEG and eye-gaze features to predict RAS skill levels. Objective skill classification models in clinical settings can improve RAS surgical training processes by providing surgeons instant feedback on their level of expertise, while they are practicing. Surgeons can immediately identify areas of improvement and adjust their training accordingly. Integration of these models into surgical training programs could lead to better skill acquisition and ultimately improve patient outcomes.

### Supplementary Information

Below is the link to the electronic supplementary material.

Supplementary file1 (DOCX 18 KB)

## Data Availability

Data supporting the findings of this study are available from the corresponding author (SBS) upon reasonable request.
