# Supplementary material for: Surgical skill level classification model development using EEG and eye-gaze data and machine learning algorithms
Source: J Robot Surg. 2023 Oct 21;17(6):2963–71. doi: 10.1007/s11701-023-01722-8 (PMC10678814; doi:10.1007/s11701-023-01722-8)
Supplement: Supplementary file 1 — Supplementary file1 (DOCX 18 KB) [file 11701_2023_1722_MOESM1_ESM.docx]

**Supplement 1:** Hyperparameters that were considered in training machine learning models in this study.

**Gradient Boosting:** It is an ensemble learning algorithm that combines multiple weak learners (decision trees) to create a strong learner. The key idea of gradient boosting is to fit each new decision tree to the residual errors made by the previous tree. In this way, the new trees complement the previous ones, gradually improving the overall prediction accuracy. The main hyperparameters for Gradient Boosting and values considered for each parameter for tuning were explained here.

‘n_estimators’: This parameter defines the number of decision trees to be used in the ensemble. A higher value of ‘n_estimators’ can lead to better performance, but also longer training times and risk of overfitting. Range in this study: 25 to 300 with increment 25.

‘learning_rate’: This parameter determines the contribution of each decision tree to the final prediction. A smaller learning rate means each tree has less influence and requires more trees to achieve the same performance. Range in this study: 0.2 to 1 with range 0.2.

‘max_depth’: This parameter defines the maximum depth of each decision tree in the ensemble. A deeper tree can learn more complex patterns but can also be overfit to the training data. Range in this study: 1 to 50 with increment 2.

‘max_features’: This parameter defines the maximum number of features to consider when looking for the best split. A smaller value can reduce overfitting but may also reduce the performance. Values in this study: 'sqrt','log2', 20, 40, 60, 80, 100. If 'sqrt' is used, the maximum number of features to be considered is the square root of the total number of features, while if 'log2' is used, the maximum number of features to be considered is the base-2 logarithm of the total number of features. For example, if a dataset has 16 features, using 'sqrt' for max_features would consider a maximum of 4 features at each split, while using 'log2' would consider a maximum of 4 or 5 features, depending on how the logarithm is calculated.

**Random Forest:** Random Forest Classifier is an ensemble learning method that combines multiple decision trees to make a final prediction. The main hyperparameters for Random Forest and values considered for each parameter for tuning were:

'n_estimators': same definition and range as gradient boosting

'criterion': This parameter used to measure the quality of a split. The two supported criteria are the Gini impurity and entropy.

'max_depth': same definition and range as gradient boosting

'max_features': same definition and range as gradient boosting

'min_samples_leaf': The minimum number of samples required to be at a leaf node. Range in this study: 1 to 5 with increment 1.

'min_samples_split': The minimum number of samples required to split an internal node. Range in this study: 1 to 10, with increment 2.

**Multinomial Logistic Regression.** Logistic regression is a linear classification algorithm used to model the probability of a certain class or event. It estimates the probability of an instance belonging to a particular class using a logistic function. The main hyperparameters for logistic regression and values considered for each parameter for tuning were explained here.

Penalty determines the type of regularization. Regularization is a technique used to avoid overfitting by adding a penalty term to the loss function. The penalty term can be 'l1', 'l2', or 'elasticnet'.

C is the inverse of regularization strength. A smaller value of C results in stronger regularization, and a larger value of C results in weaker regularization.

Solver is an optimization algorithm used to optimize the loss function. The 'newton-cg', 'lbfgs', 'sag', or 'saga' were considered for this parameter.

Hyperparameters were tuned using grid search technique, where a range of values was explored, and the best combination was selected based on a performance metric.
